# Supplementary material for: Single cell spatial analysis reveals the topology of immunomodulatory purinergic signaling in glioblastoma
Source: Nat Commun. 2022 Aug 16;13:4814. doi: 10.1038/s41467-022-32430-w (PMC9381513; doi:10.1038/s41467-022-32430-w)
Supplement: Supplementary file 1 — Supplementary Information [file 41467_2022_32430_MOESM1_ESM.pdf]

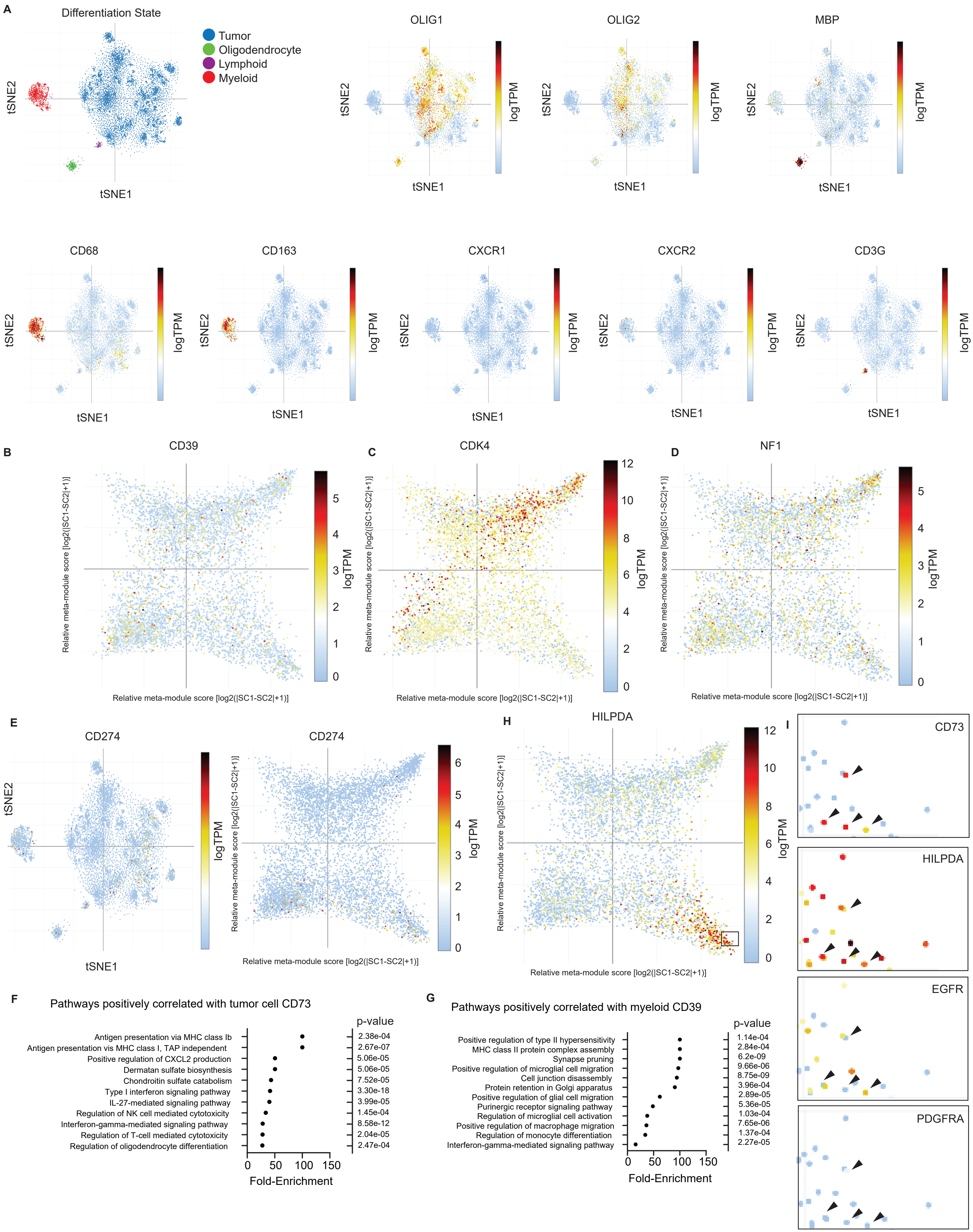
A
Differentiation State
Tumor
Oligodendrocyte
Lymphoid
Myeloid
tSNE1
tSNE2
OLIG1
OLIG2
MBP
CD68
CD163
CXCR1
CXCR2
CD3G
logTPM
B
CD39
C
CDK4
D
NF1
Relative meta-module score [log2(|SC1-SC2|+1)]
E
CD274
H
HILPDA
I
CD73
HILPDA
EGFR
PDGFRA
F
Pathways positively correlated with tumor cell CD73
p-value
Antigen presentation via MHC class Ib 2.38e-04
Antigen presentation vis MHC class I, TAP independent 2.67e-07
Positive regulation of CXCL2 production 5.06e-05
Dermatan sulfate biosynthesis 5.06e-05
Chondroitin sulfate catabolism 7.52e-05
Type I interferon signaling pathway 3.30e-18
IL-27-mediated signaling pathway 3.99e-05
Regulation of NK cell mediated cytotoxicity 1.45e-04
Interferon-gamma-mediated signaling pathway 8.58e-12
Regulation of T-cell mediated cytotoxicity 2.04e-05
Regulation of oligodendrocyte differentiation 2.47e-04
Fold-Enrichment
G
Pathways positively correlated with myeloid CD39
p-value
Positive regulation of type II hypersensitivity 1.14e-04
MHC class II protein complex assembly 2.84e-04
Synapse pruning 6.2e-09
Positive regulation of microglial cell migration 9.66e-06
Cell junction disassembly 8.75e-09
Protein retention in Golgi apparatus 3.96e-04
Positive regulation of glial cell migration 2.89e-05
Purinergic receptor signaling pathway 5.36e-05
Regulation of microglial cell activation 1.03e-04
Positive regulation of macrophage migration 7.65e-06
Regulation of monocyte differentiation 1.37e-04
Interferon-gamma-mediated signaling pathway 2.27e-05
Fold-Enrichment

Supplementary Figure 1.

(**A**) Evaluation of additional lineage-specific markers in single-cell RNA-sequencing data shows expression of *OLIG1*, *OLIG2*, and *MBP* in oligodendrocyte populations; *CD68*, *CD163* in myeloid populations; *CD3G* in lymphoid populations. CXCR1 and CXCR2 showed no significant neutrophil population. (**B**) Lineage analysis of tumor cells shows low-level expression of *CD39* in tumor cells, with no apparent enrichment in any lineage group. (**C, D**) *CDK4* showed appropriate localization to the NPC-like cluster, while *NF1* showed slightly lower signal in the MES-like cluster. (**E**) PD-L1 (*CD274)* was expressed only at very low levels in glioblastoma, with slight enrichment in the AC-like and MES-like clusters. (**F**) GSEA analysis of tumor cell populations showed that chemokine (CXCL2), major histocompatibility (MHC-I) and interferon signaling are the pathways most strongly correlated with high *CD73* expression (GSEA, Kolmogorov-Smirniov test, unpaired, one-sided). (**G**) GSEA analysis of myeloid cells showed that in addition to purinergic signaling, inflammatory pathways including MHC-II and interferon signaling, and those regulating microglial migration and activation are the most upregulated in cells with high *CD39* expression (GSEA, Kolmogorov-Smirniov test, unpaired, one-sided). (**H**) The hypoxia-responsive gene *HILPDA* was strongly expressed in the MES-like cluster, but not other lineages. (**I**) Close-inspection of rare single-cells with elevated *CD73* in the MES-like cluster showed co-localization with strong *HILPDA* expression.

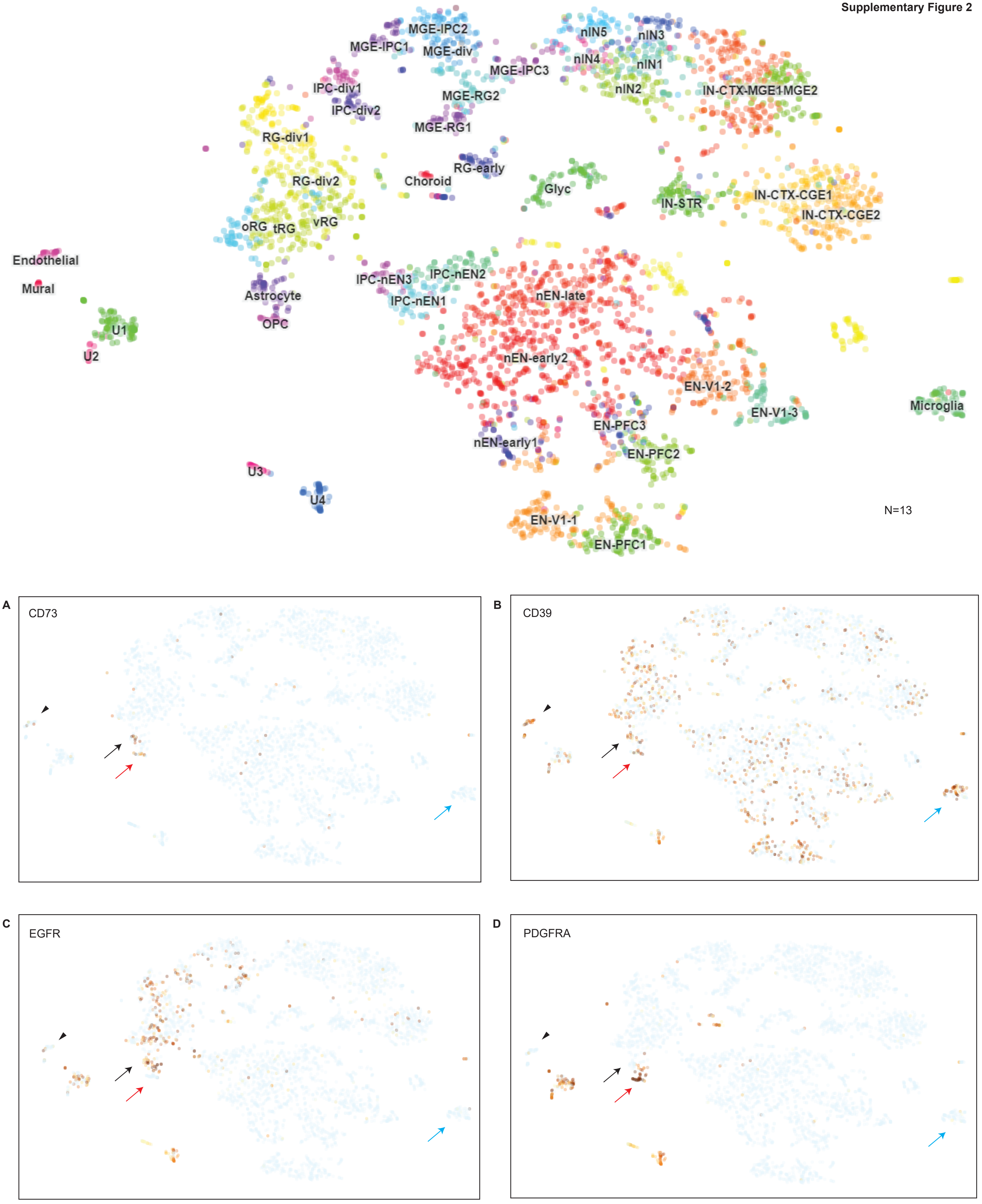
MGE-IPC2
MGE-IPC1
MGE-div
MGE-IPC3
IPC-div1
IPC-div2
MGE-RG2
MGE-RG1
RG-div1
RG-div2
RG-early
Choroid
Glyc
oRG
tRG
vRG
Endothelial
Mural
Astrocyte
OPC
U1
U2
U3
U4
IPC-nEN3
IPC-nEN2
IPC-nEN1
nEN-late
nEN-early2
nEN-early1
nIN5
nIN3
nIN4
nIN1
nIN2
IN-CTX-MGE1
MGE2
IN-STR
IN-CTX-CGE1
IN-CTX-CGE2
EN-V1-2
EN-V1-3
EN-PFC3
EN-PFC2
EN-V1-1
EN-PFC1
Microglia
N=13
A
CD73
B
CD39
C
EGFR
D
PDGFRA

Supplementary Figure 2.

(**A**) Analysis of non-neoplastic human developing cortex (UCSC Cell Browser) showed that *CD73* is predominantly enriched in developing astrocytes (black arrow) and OPC (red arrow). (**B**) *CD39* showed a broader expression distribution but was more strongly expressed by microglia (blue arrow) and endothelial cells (arrowhead). (**C,D**) *EGFR* was strongly expressed by developing astrocytes (black arrow) but not OPC (red arrow) while *PDGFRA* was enriched in OPC (red arrow) but not developing astrocyte (see https://cells.ucsc.edu/?ds=cortex-dev for further classification of cell subtypes).

Supplementary Figure 3

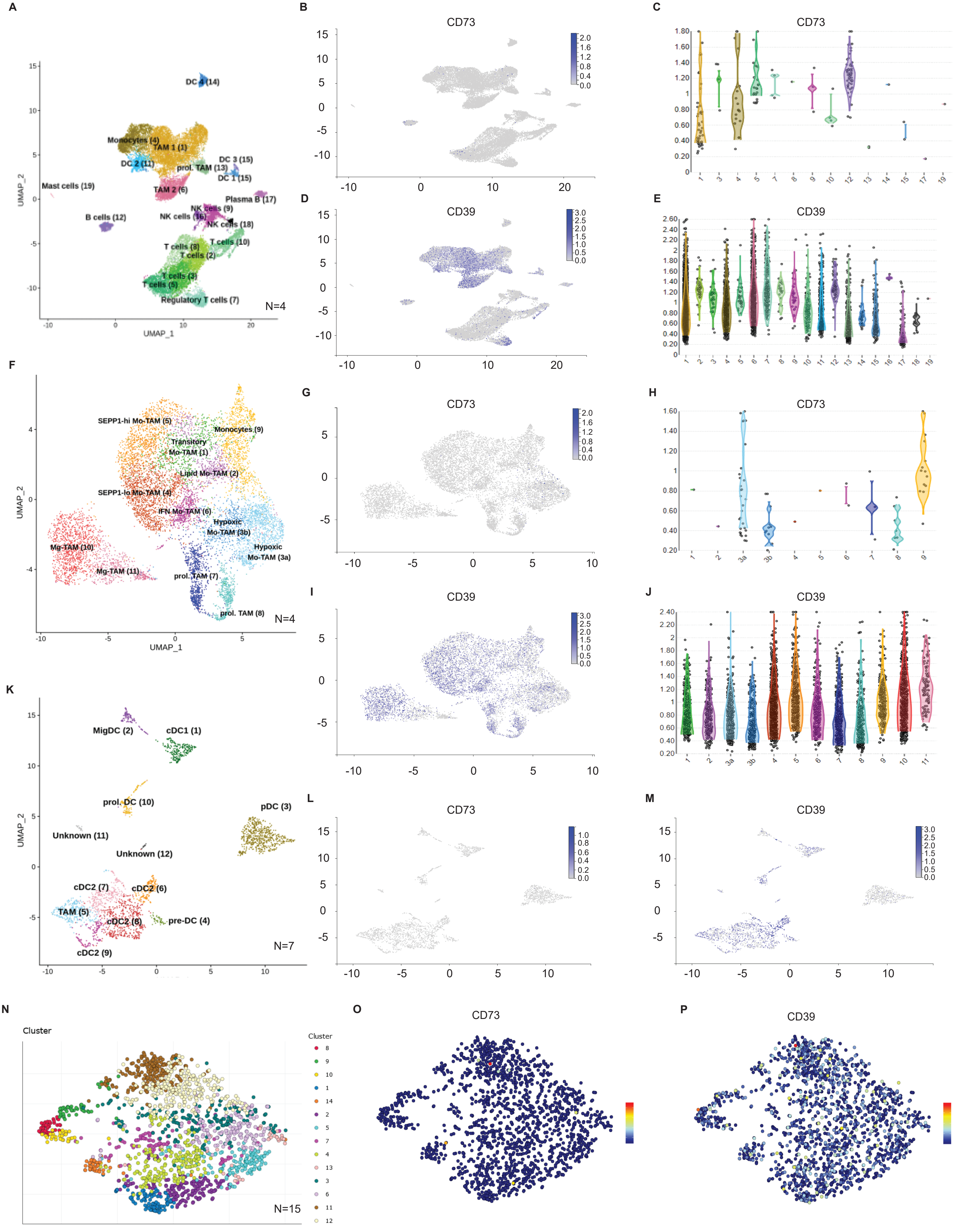

Supplementary Figure 3.

(**A**) Single-cell analysis FACS-sorted CD45-positive immune cells from 4 recurrent adult IDH-WT glioblastomas (Pombo Antunes et al.) (n=42,870), showed only very rare *CD73* expression (0.7% of cells), predominantly in TAM and monocytes. (**B,C**) In contrast to newly-diagnosed tumors, there is a greater number of *CD73*-high T and B cells in recurrent tumors. (**D**,**E**) *CD39* expression is present in all immune populations, though it is most enriched in TAM1 and TAM2 clusters. There is a slightly reduced overall proportion of *CD39*-positive immune cells in recurrent cases, likely due to an influx of *CD39*-negative T cells, though there is a new prominent regulatory T cell (Treg) cluster with strong *CD39* expression in recurrences compared to newly-diagnosed tumors. (**F-H**) TAM sub-group analysis showed low overall *CD73* expression, though hypoxic monocytic TAM clusters are most enriched for *CD73*. (**I,J**) Sub-group analysis of *CD39* expression showed broad expression in all TAM sub-groups, with the greatest enrichment in microglial TAM clusters. (**K,L**) *CD73* was not expressed in any dendritic cell population (0% of cells). (**M**) *CD39* expression was low in plasmacytoid and migratory dendritic cells, but broadly expressed by other dendritic cell sub-groups. (**N-P**) Single-cell RNA-sequencing analysis of an additional cohort of glioma-associated microglial cells (Sankowski *et al.*) showed minimal expression of *CD73* in all microglial sub-groups, while *CD39* expression was present in all sub-groups.

Supplementary Figure 4

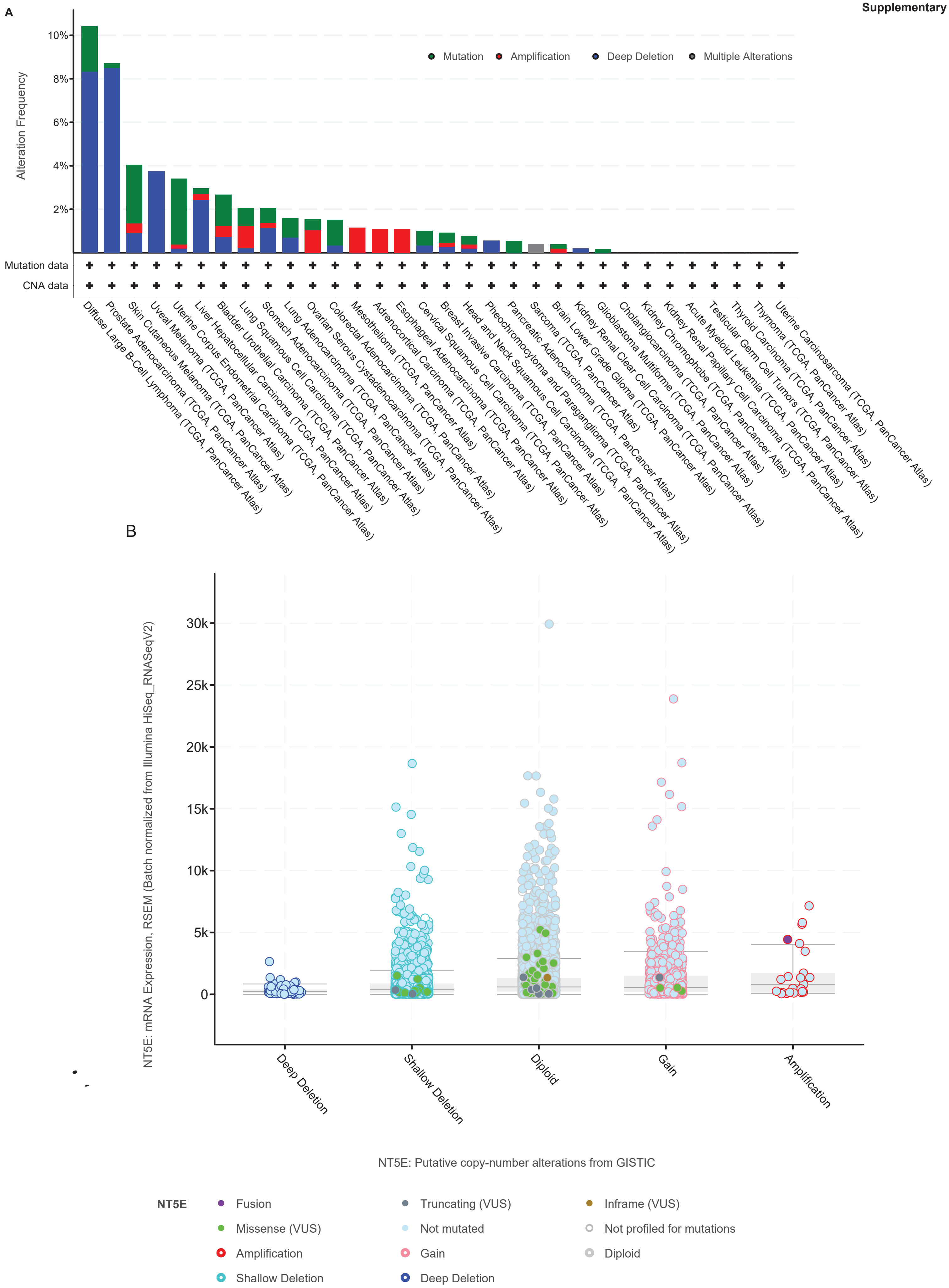

Supplementary Figure 4.

(**A**) *CD73* gene alterations are uncommon in cancer (TCGA Pan-Cancer Atlas, n=32 tumor types, 10,967 samples), and present in <1% of glioblastomas. (**B**) *CD73* mRNA expression levels are not significantly correlated with underlying alteration of the *CD73* gene in glioblastoma (n=145), with no elevation in expression in *CD73* amplified tumors.

Supplementary Figure 5

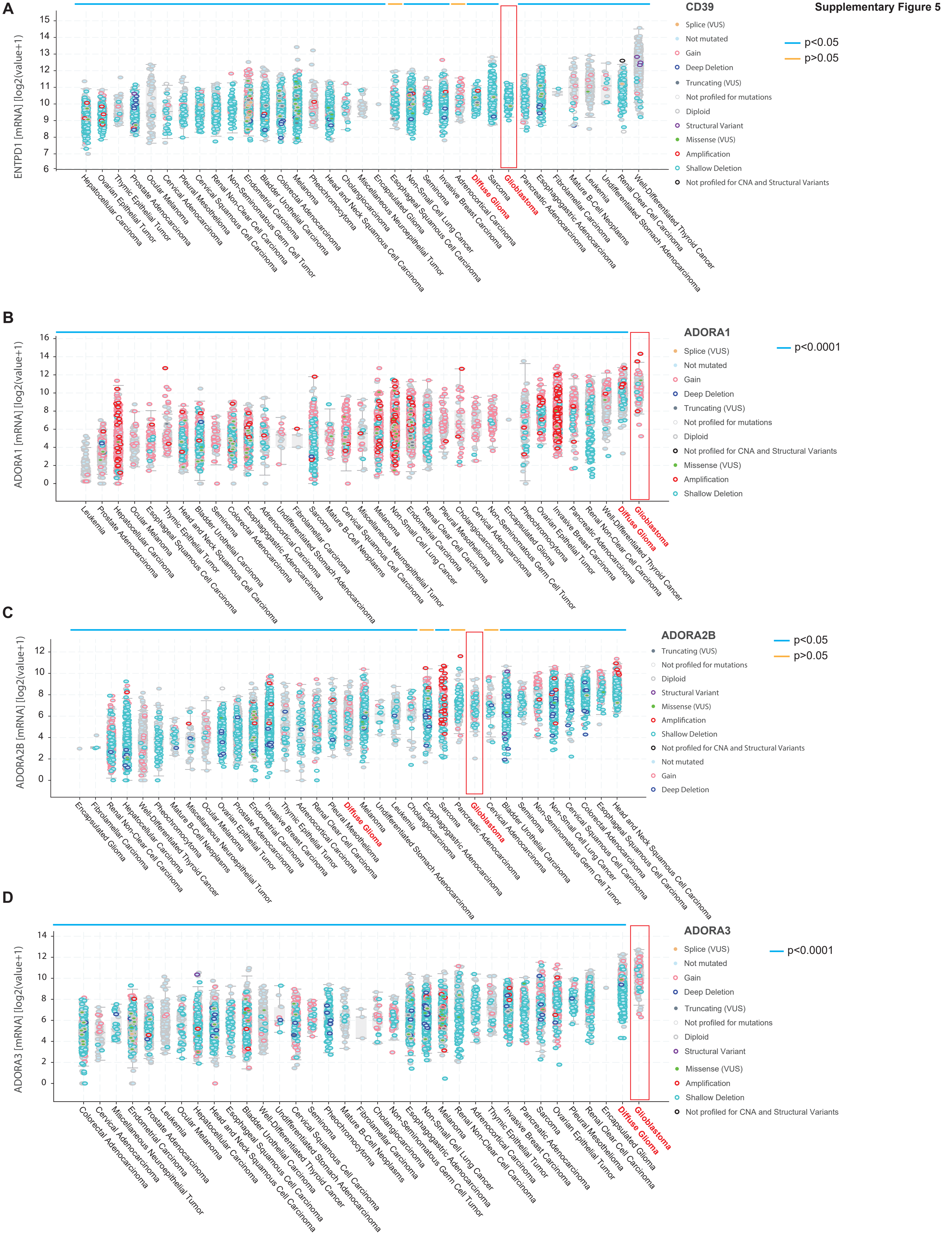

Supplementary Figure 5.

(**A**) Bulk mRNA expression was assessed in tumor tissue (n=10,967) from 32 cancer subtypes (TCGA) (see Supplementary Table S4 for detailed information including specimen counts and precise p-values for each comparison depicted in A-D). *CD39* exhibits significantly higher expression in glioblastoma than many cancer subtypes ($p<0.05$, blue line, t-test, unpaired, two-sided, with Welch's correction). (**B-D**) Analysis of adenosine receptors shows that glioblastomas and diffuse gliomas demonstrate higher *ADORA1* and *ADORA3* expression than all other cancer subtypes ($p<0.0001$), and relatively high levels of *ADORA2B* compared to many cancers (t-test, unpaired, two-sided, with Welch's correction). *ADORA2A* expression is relatively less enriched in glioblastoma (Table S4, not shown). (Figure S5A-D use Tukey box-and-whisker plots with midline = median, box limits = Q1 (25th percentile)/Q3 (75th percentile), whiskers = 1.5 inter-quartile range (IQR), with individual tumor values also superimposed on the graph with relevant genotypic alterations noted in the color code).

Supplementary Figure 6

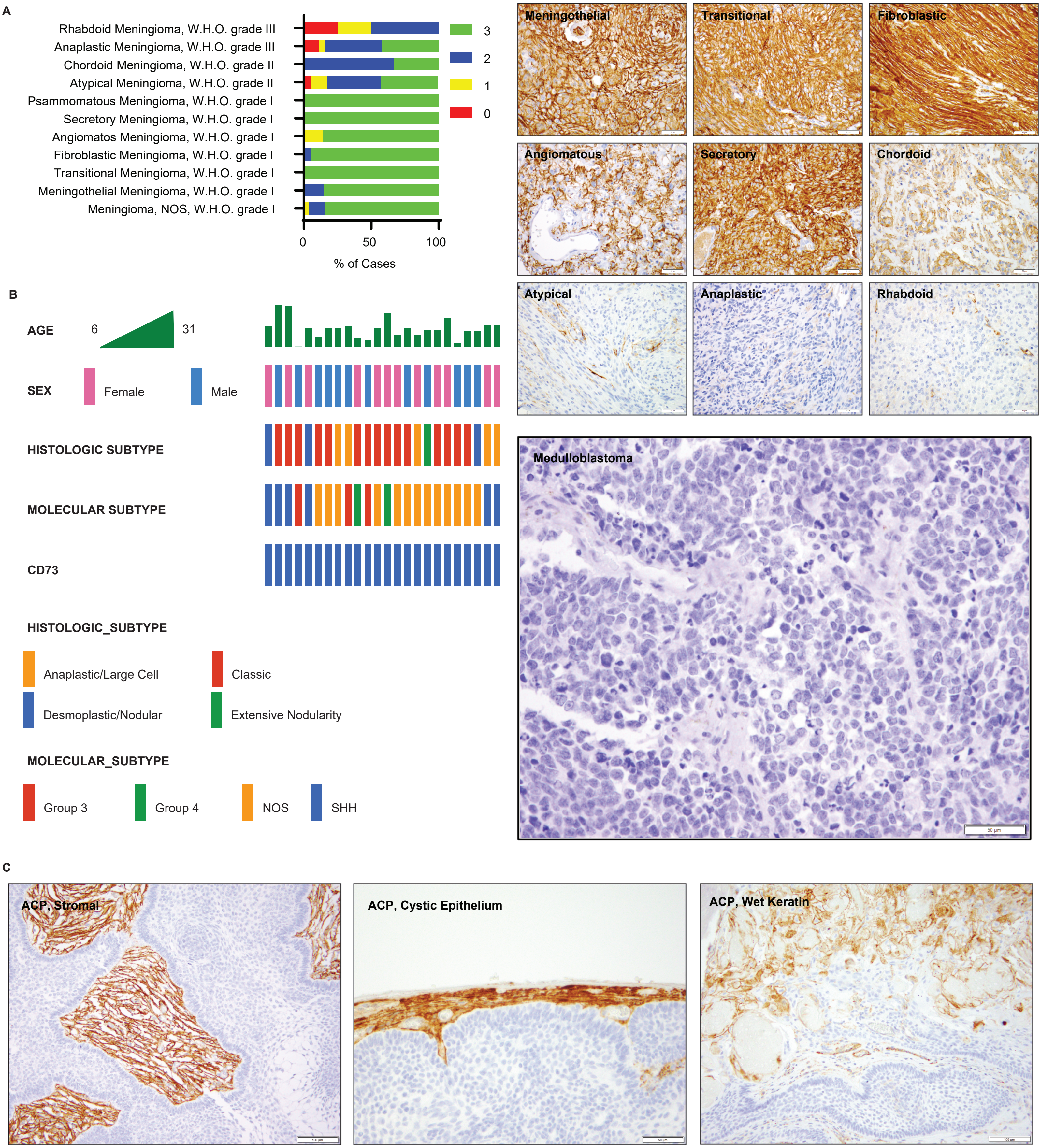

Supplementary Figure 6.

(**A**) Extended immunohistochemistry analysis shows that W.H.O. grade 1 meningiomas exhibit consistently high CD73 expression in all histologic subtypes (each specimen stained once). Lower CD73 expression is observed in atypical and chordoid meningiomas (W.H.O. grade 2), with the lowest levels in high-grade anaplastic and rhabdoid meningiomas (W.H.O. grade 3); scalebars: 50µm. (**B**) Medulloblastomas exhibited no CD73 expression regardless of histologic or molecular subtype; scalebar: 50µm. (**C**) Adamantinomatous craniopharyngiomas exhibited regional variations in CD73 expression, with no expression in the basaloid, stellate reticular, or whorled tumor epithelium, but consistently strong expression in the fibrovascular stroma, keratinized cyst-lining epithelium, and regions of wet keratin. Representative staining of specimens from each tumor subtype are shown; each immunohistochemistry staining experiment was performed once; scalebars: 100µm.

Supplementary Figure 7

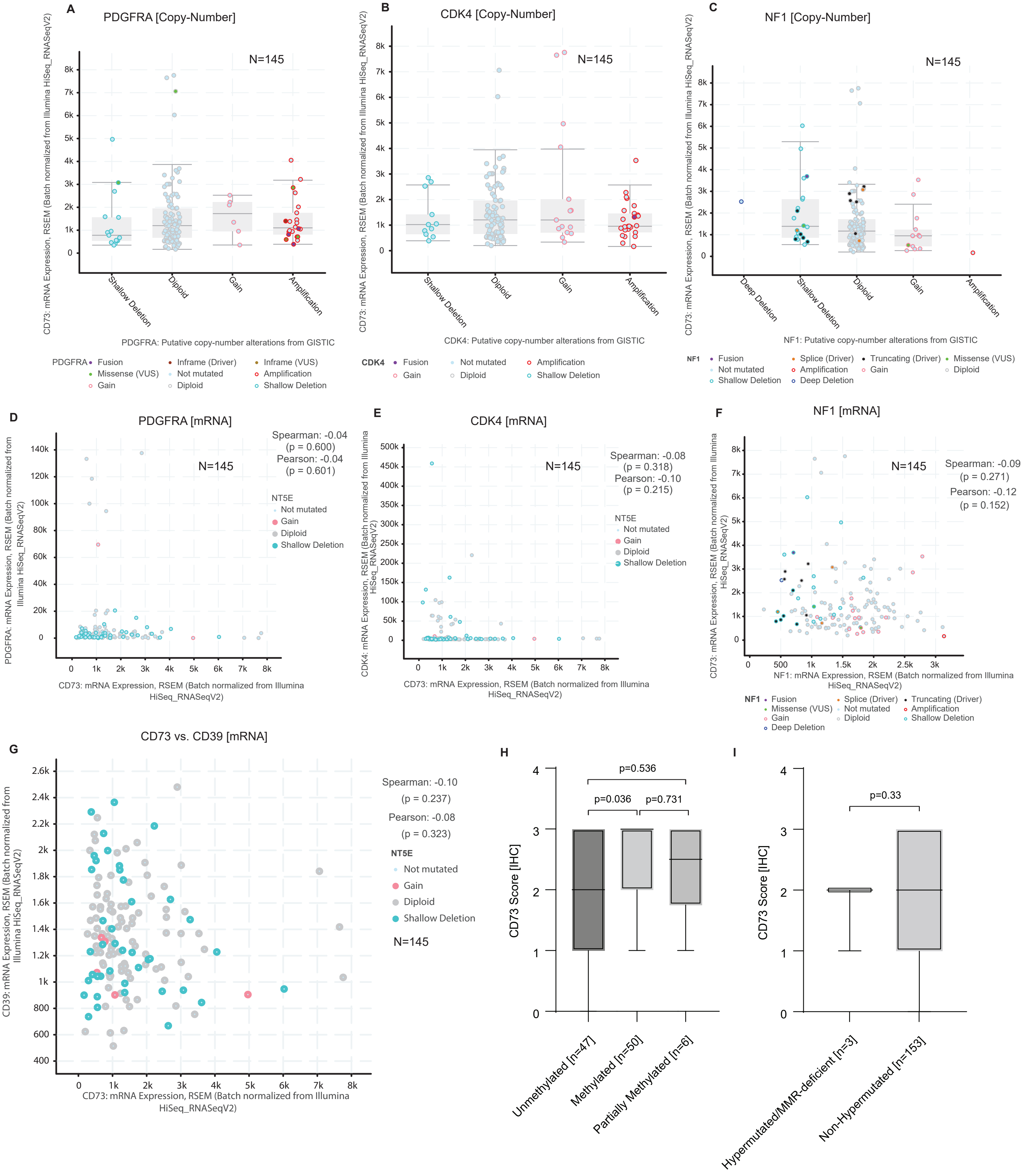

Supplementary Figure 7.

(**A-C**) Bulk mRNA analysis of human glioblastoma specimens (TCGA, n=145 tumors) shows no evident correlation between *CD73* expression level and chromosomal alterations in *PDGFRA*, *CDK4*, or *NF1*. (**D-F**) There was no significant correlation between mRNA expression of *CD73* and *PDGFRA* (p=0.60, Pearson correlation test, unpaired, two-sided), *CDK4 (*p=0.22, Pearson correlation test, unpaired, two-sided), or *NF1* (p=0.15, Pearson correlation test, unpaired, two-sided). (**G**) There was no significant correlation between tumor *CD73* and *CD39* levels (p=0.32, Pearson correlation test, unpaired, two-sided). (**H**) Mean CD73 IHC expression was slightly lower in tumors with an unmethylated *MGMT* promoter (n=47 tumors), compared to those with methylation of this locus (n=50 tumors) (p=0.038, t-test, unpaired, two-sided). (**I**) There was no significant difference in CD73 IHC expression in recurrent/residual glioblastomas with hypermutation and mismatch-repair deficiency (n=3 tumors) from those without (n=153 tumors) (p=0.33, t-test, unpaired, two-sided), though the number of hypermutated tumors in the cohort was small. (Figures SA-C, H ,and I use Tukey box-and-whisker plots with midline = median, box limits = Q1 (25th percentile)/Q3 (75th percentile), whiskers = 1.5 inter-quartile range (IQR), dots = outliers (>1.5IQR)).

Supplementary Figure 8

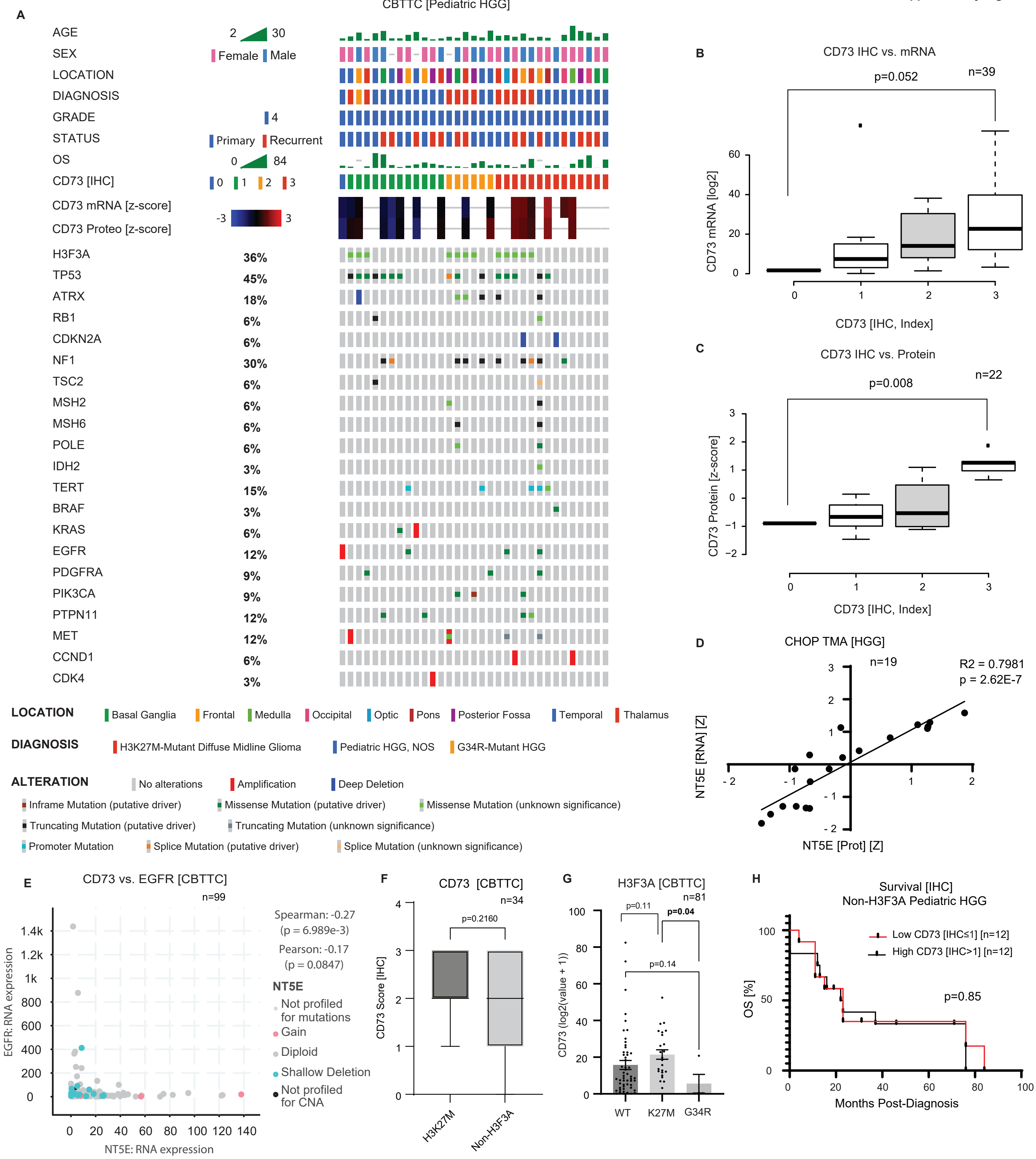

Supplementary Figure 8.

(**A**) Sub-group analysis of a cohort of 34 pediatric HGG from the Children's Brain Tumor Network (CBTN) dataset with curated clinical and molecular data showed a wide variety of molecular alterations, with fewer alterations per case that adult tumors, consistent with the typical genomic diversity of pediatric gliomas. H3K27M-mutant diffuse midline gliomas and pediatric HGG frequently exhibit high expression of CD73 (IHC). (**B**) CD73 IHC score showed a trend toward correlation with *CD73* mRNA on evaluation of all HGG with mRNA data (n=39), but this did not reach statistical significance in all dataset (p=0.052, Kruskall-Wallis test, unpaired, two-sided). (**C**) CD73 IHC score was strong correlated with CD73 protein levels by mass spectrometry (p=0.008, n=22, Kruskall-Wallis test, unpaired, two-sided). (**D**) There was a strong correlation between CD73 mRNA and protein levels in tumors in which both datasets were available (p=2.62e-7, n=19, t-test, unpaired, two-sided). (**E**) Bulk mRNA sequencing from the full CBTTC dataset (n=99 tumors) showed a negative correlation between *CD73* and *EGFR* levels (p=0.08, Pearson correlation test, unpaired, two-sided). (**F**) Comparison of H3K27M-mutant diffuse midline glioma and non-H3F3A mutated pediatric HGG showed no significant difference in CD73 expression, with both exhibiting high mean CD73 (n=34, p=0.20, t-test, unpaired, two-sided); (**G**) however, H3G34R-mutant hemispheric gliomas exhibited significantly lower levels of *CD73* (n=81 total tumors, p=0.04, t-test, unpaired, two-sided; column plot, mean±S.E.M.). (**H**) Unlike adult IDH-WT glioblastoma, non-H3F3A-mutated pediatric HGG showed no difference in survival between cases with high *CD73* (top 50% of expression) and low *CD73* (bottom 50% of expression) (p=0.85, Mantel-Cox log-rank). (Figure S8B,C,F use Tukey box-and-whisker plots with midline = median, box limits = Q1(25th percentile)/Q3 (75th percentile), whiskers = 1.5 inter-quartile range (IQR), dots = outliers(>1.5IQR))

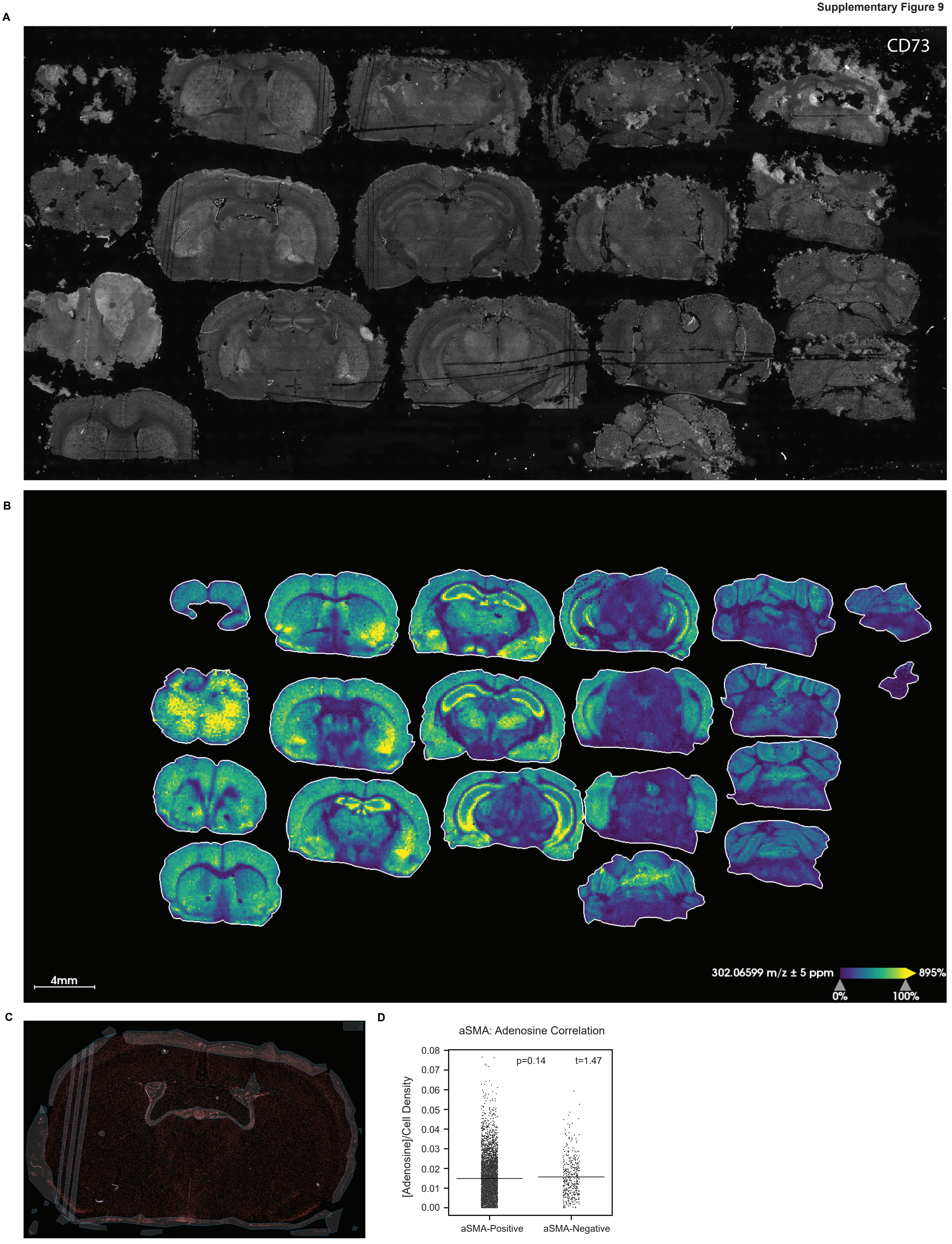
A
CD73
B
4mm
302.06599 m/z ± 5 ppm
895%
0%
100%
C
D
aSMA: Adenosine Correlation
p=0.14
t=1.47
[Adenosine]/Cell Density
0.08
0.07
0.06
0.05
0.04
0.03
0.02
0.01
0.00
aSMA-Positive
aSMA-Negative

Supplementary Figure 9.

(**A**) CD73 immunofluorescence in serial coronally sectioned whole-mount mouse brain showed enrichment of CD73 protein in the basal ganglia, with weaker expression in the cerebral and cerebellar cortex. There was minimal expression in the central white matter tracts and brain-stem. (**B**) Spatially-resolved mass-spectrometry imaging showed a similar distribution of adenosine, with enrichment predominantly in the basal ganglia, with somewhat weaker signal in the cortex and minimal levels in the white matter tracts and brainstem; scale bar, 4mm. (**C**) During registration and processing of CD73 immunofluorescence and adenosine imaging, tissue tears and artifacts were identified, segmented, and removed from analysis to improve signal-to-noise. (**D**) While there was a strong spatial correlation between CD73 and adenosine levels, no such correlation was present for αSMA, which labels vascular smooth muscle ($p=0.14$, $t=1.47$, Tukey's HSD, unpaired, one-sided).

Supplementary Figure 10

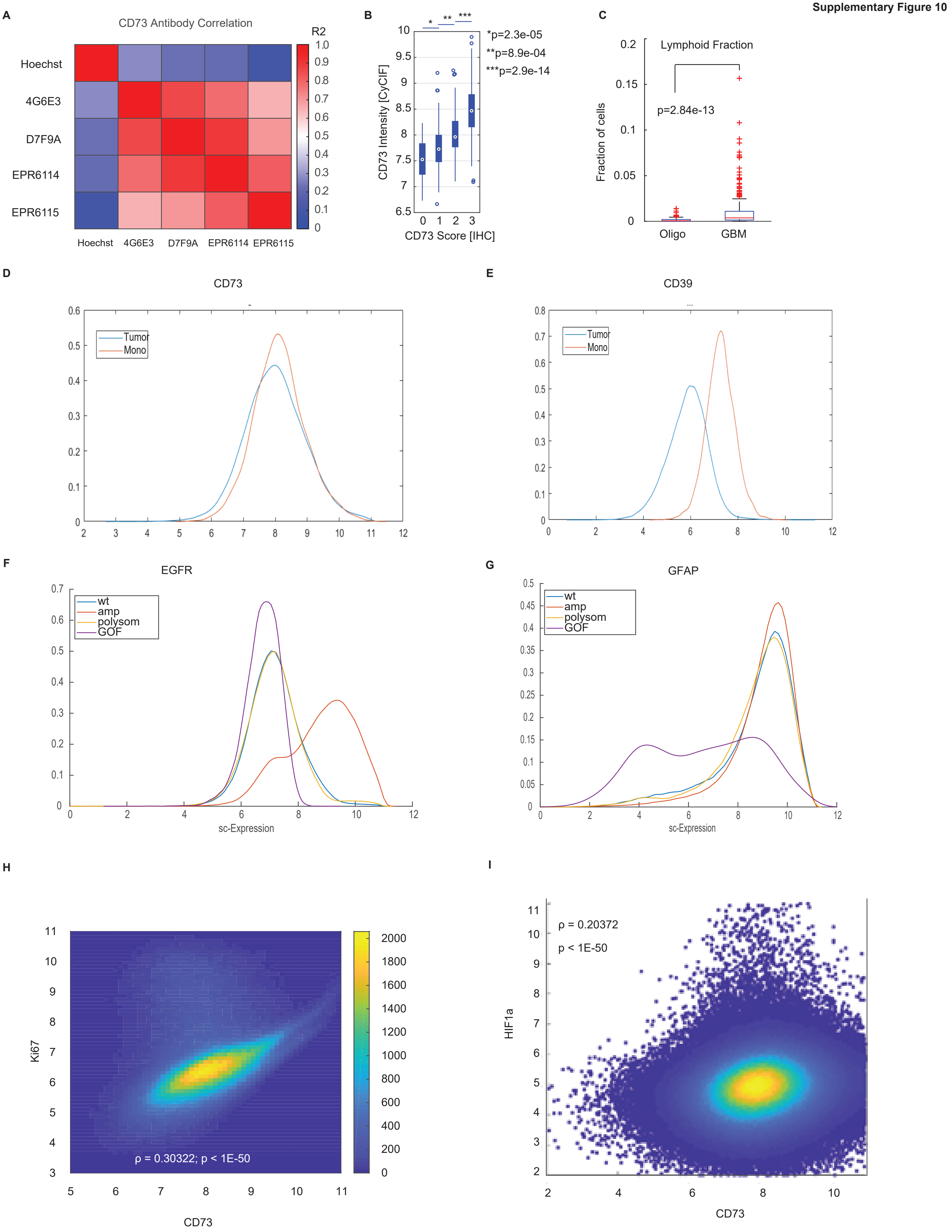

Supplementary Figure 10.

(**A**) Cyclic immunofluorescence was used to correlate CD73 signal across all 172 IDH-WT glioblastoma samples using multiple CD73 antibody clones, showing strong pixel-level correlation between all clones, but not unrelated markers such as nuclear Hoechst. (**B**) There was an excellent correlation between CD73 IHC index score (as shown in Fig. 4), and mean core-level tumor cell CD73 intensity levels quantitatively measured by CyCIF (n=172 tumors, pairwise comparisons performed by t-tests, unpaired, two-sided). (**C**) Single-cell immune profiling of cells from the 172 IDH-WT CyCIF dataset showed that lymphoid cells are uncommon in glioblastoma, often comprising <5% of cells. Lymphoid populations are even less common in low grade oligodendrogliomas (n=14) analyzed in this dataset (p=2.84e-13, Wilcoxon rank sum test, unpaired, two-sided). (**D**) CD73 expression is present in both tumor and myeloid (mono) cells in the CyCIF IDH-WT dataset at similar levels, though the number of CD73-positive tumor cells is greater (**Fig. 7**). (**E**) CD39 is much more strongly expressed by myeloid (mono) populations than tumor cells in IDH-WT Glioblastoma. (**F-G**) Single-cell analysis of functional markers in IDH-WT glioblastoma (n=172) showed that CD73 levels are enriched in tumors with underlying EGFR amplification, and associates with high levels of GFAP. (**H,I**) Single-cell analysis showed that CD73 is positively correlated with the proliferation marker ki-67/MIB-1 (p<1e-50, t-test on Z-transformed Pearson correlation, unpaired, two-sided), and HIF1A (p<1e-50, t-test on Z-transformed Pearson correlation, unpaired, two-sided). (Figures S10B and S10C use Tukey box-and-whisker plots with midpoint = median, box limits = Q1(25th percentile)/Q3 (75th percentile), whiskers = 1.5 inter-quartile range (IQR), dots = outliers(>1.5IQR))

Supplementary Figure 11

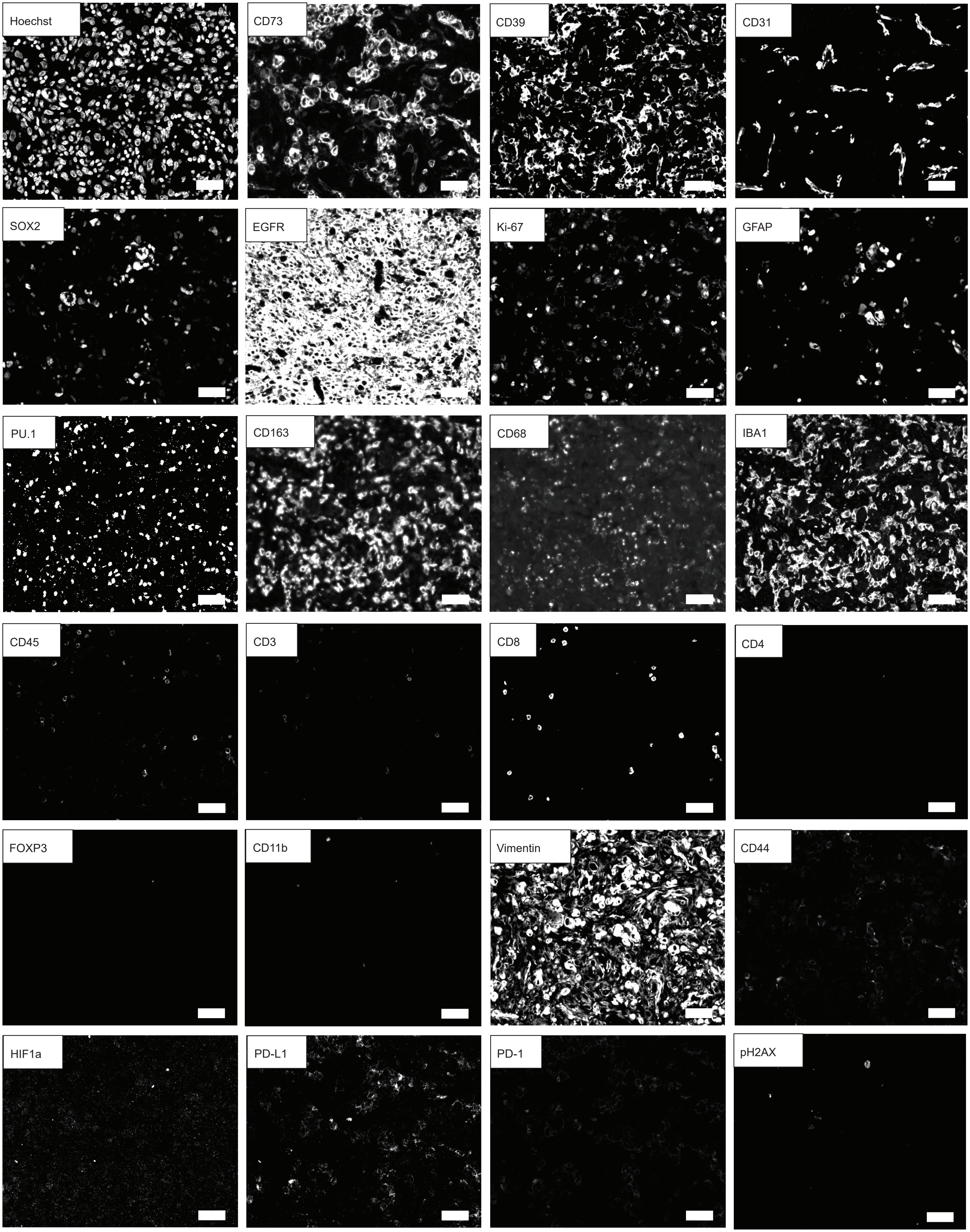

Supplementary Figure 11.

Tissue-based cyclic immunofluorescence (CyCIF) was performed on a tissue microarray (TMA) containing 172 IDH-WT glioblastoma specimens. Representative images of the same field from a glioblastoma specimen for each marker are shown, CyCIF was performed once on each specimen; scalebars: 50μm.

Supplementary Figure 12

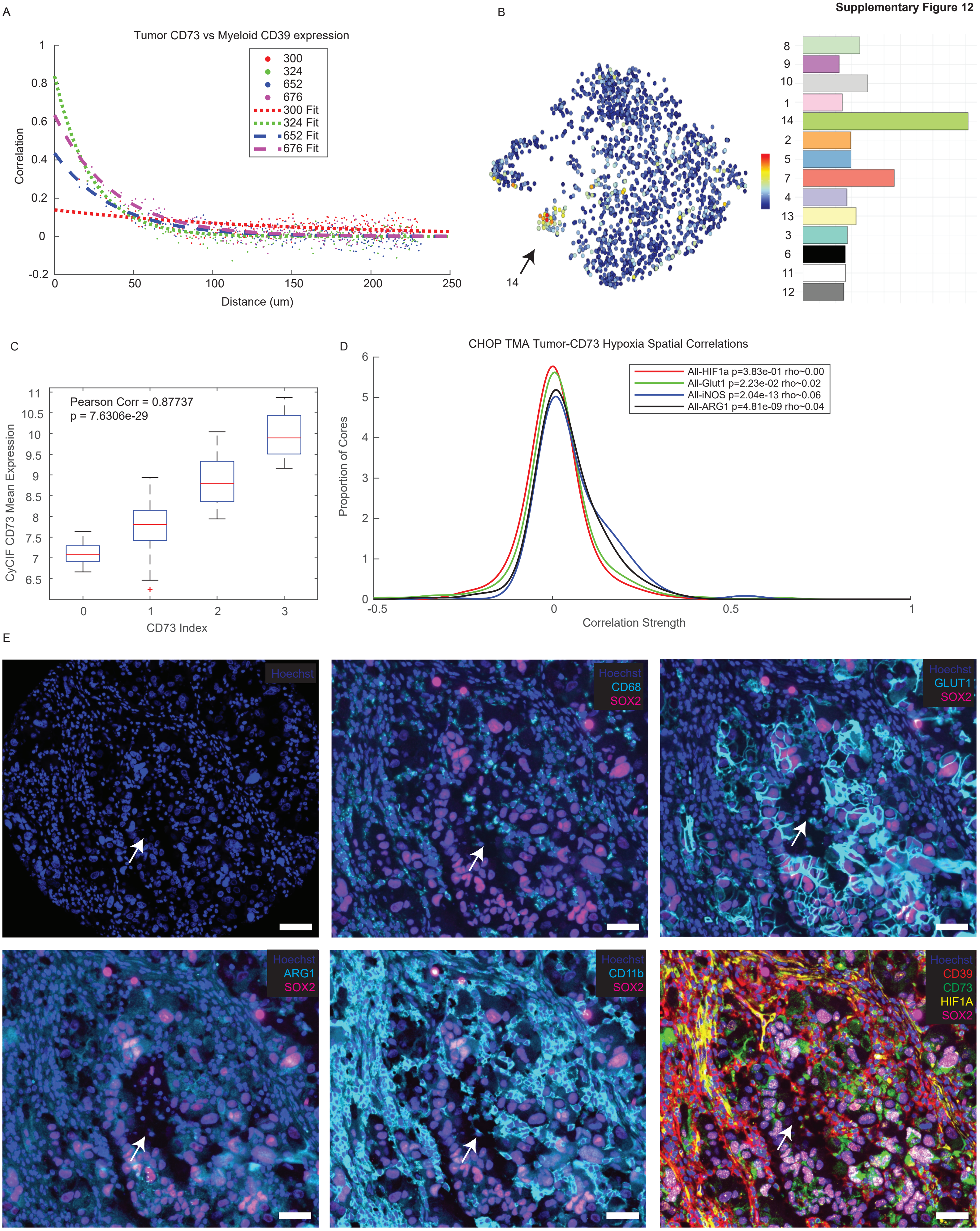

Supplementary Figure 12.

(**A**) Spatial statistics correlation analysis of a representative glioblastoma specimen shows a strong spatial correlation between tumor CD73 and myeloid CD39 expression in a representative case. (**B**) Microglial sub-group analysis (Sankowski et al.[52]) of the myeloid group (CD39, CD11b, CD163) associated with tumor CD73 in IDH-WT glioblastoma shows that this signature strongly correlates with 'cluster 14', a group of glioma-associated microglia associated with inflammation and hypoxia signaling. (**C**) CD73 IHC score and mean CD73 intensity by CyCIF (all cells) were strongly correlated in the CBTTC pediatric glioma dataset (n=87 tissue cores, p=7.6e-29, Pearson's correlation test, unpaired, two-sided). (**D**) Spatial statistics analysis of the pediatric glioma dataset showed a significant correlation between tumor CD73 and hypoxia-responsive genes GLUT1 (p=2.23e-2), iNOS (p=2.0e-13), and ARG1 (p=4.8e-9), but not HIF1A (p=0.38, Pearson's correlation tests, unpaired, two-sided). (**E**) Analysis of CyCIF staining showed that these markers are sometimes elevated in regions of strong tumor CD73 and myeloid CD39 co-localization, which may be associated with regions of palisading necrosis (**arrow**) (representative staining from a high-grade glioma specimen is shown, CyCIF was performed once on each specimen); scalebars: 50µm. (Figure S12C uses a Tukey box-and-whisker plot with midline = median, box limits = Q1(25th percentile)/Q3 (75th percentile), whiskers = 1.5 inter-quartile range (IQR), dots = outliers(>1.5IQR))

Supplementary Table 1: Antibody Information

Target	Clone #	Vendor	Catalog #	Dilution	RR_ID
anti-Arginase-1	D4E3M	Cell Signaling Technology	66297	1:100	AB_2799705
anti-CA IX	H-11	Santa Cruz Biotechnology	sc-365900	1:100	AB_10846466
anti-CD11b	C67F154	eBioscience -Thermo Fisher Scientific	53-0196-80	1:200	AB_2637195
anti-CD11b	C67F154	eBioscience -Thermo Fisher Scientific	53-0196-82	1:100	AB_2637195
anti-CD14	EPR3653	Abcam	ab196169	1:800	AB_2890135
anti-CD163	EPR14643-36	Abcam	ab218293	1:100	AB_2889155
anti-CD206	D-1	Santa Cruz Biotechnology	sc-376108	1:100	AB_10987732
anti-CD24	SN3	Abcam	ab30350	1:200	AB_726282
anti-CD3	CD3-12	Abcam	ab11089	1:500	AB_2889189A
anti-CD31	EPR3094	Abcam	ab218582	1:500	AB_2857973
anti-CD39	EPR20627	Abcam	ab236038	1:100	n/a
anti-CD4	N1UG0	eBioscience -Thermo Fisher Scientific	41-2444-82	1:100	AB_2573602
anti-CD40	D8W3N	Cell Signaling Technology	40868	1:100	AB_2799188
anti-CD44	EPR1013Y	Abcam	ab194987	1:100	n/a
anti-CD44	156-3C11	Cell Signaling Technology	8724	1:200	AB_10829611
anti-CD45	HI30	BioLegend	304039	1:200	AB_2562057
anti-CD68	D4B9C	Cell Signaling Technology	79594S	1:200	AB_2799935
anti-CD73	EPR6115	Abcam	ab124725	1:200	AB_10976033
anti-CD73	EPR6114	Abcam	ab133582	1:200	n/a
anti-CD73	4G6E3	Abcam	ab202122	1:200	AB_2858258
anti-CD73	D7F9A	Cell Signaling Technology		1:200	AB_2716625
anti-CD8a	AMC908	eBioscience -Thermo Fisher Scientific	50-0008-80	1:200	AB_2574148
anti-EGFR	D38B1	Cell Signaling Technology	5616	1:200	AB_10691853
anti-FOXO3A	D19A7	Cell Signaling Technology	14592	1:200	AB_2798529
anti-FOXP3	236A/E7	eBioscience -Thermo Fisher Scientific	41-4777-82	1:200	AB_2573609
anti-GFAP	GA5	eBioscience -Thermo Fisher Scientific	41-9892-82	1:100	AB_2573656
anti-GLUT1	EPR3915	Abcam	ab195020	1:100	AB_2783877
anti-HIF-1a	D1S7W	Cell Signaling Technology	52496	1:100	AB_2799414
anti-IBA1	EPR6136(2)	Abcam	ab195031	1:500	AB_2889157
anti-iNOS	EPR16635	Abcam	ab209594	1:100	AB_2889252
anti-Ki67	D3B5	Cell Signaling Technology	11882	1:400	AB_2687824
anti-MeCP2	D4F3	Cell Signaling Technology	34113	1:200	AB_2799046
anti-Mouse		Invitrogen	A-21235	1:1000	AB_2535804
anti-NeuroD1		Novus Biologicals	NBP2-98697	1:200	n/a
anti-Olig-2		Millipore	AB9610-AF555	1:200	AB_570666
anti-p21/CDKN1a	12D1	Cell Signaling Technology	8587	1:200	AB_10892861
anti-p53	E26	Abcam	ab224942	1:400	AB_2889206
anti-PD1	EPR4877(2)	Abcam	ab201825	1:200	AB_2728811
anti-PDGFRa	D13C6	Cell Signaling Technology	8893	1:200	AB_2797679
anti-PD-L1	E1L3N	Cell Signaling Technology	15005	1:200	AB_2728832
anti-pH2AX	2F3	BioLegend	613412	1:100	AB_2616871
anti-PU.1	G148-74	BD	Biosciences	1:200	AB_395335
anti-PU.1	9G7	Cell Signaling Technology	81886	1:100	AB_2799984
anti-Rabbit		Invitrogen	A-11008	1:1000	AB_143165
anti-Rabbit (labelled polymer HRP) Envision System		Dako - Agilent	K4011		
anti-Rat		Invitrogen	A-21434	1:1000	AB_141733
anti-SOX2	EPR3131	Abcam	ab196637	1:100	n/a
anti-SOX2	D6D9	Cell Signaling Technology	5179	1:200	AB_10828439
anti-Survivin	71G4B7	Cell Signaling Technology	2810	1:200	AB_10691462
