# Supplementary material for: Single cell spatial analysis reveals the topology of immunomodulatory purinergic signaling in glioblastoma
Source: Nat Commun. 2022 Aug 16;13:4814. doi: 10.1038/s41467-022-32430-w (PMC9381513; doi:10.1038/s41467-022-32430-w)
Supplement: Supplementary file 3 — Description of Additional Supplementary Files [file 41467_2022_32430_MOESM3_ESM.pdf]

Description of Additional Supplementary Data Files

File Name: Supplementary Data 1
Description: Immune cell lineage gene signatures used to analyze the tumor-immune microenvironment in glioblastoma.

File Name: Supplementary Data 2
Description: Inflammatory pathway gene signatures used to analyze the tumor-immune microenvironment in glioblastoma.

File Name: Supplementary Data 3
Description: Comparison of *CD73* bulk mRNA expression in glioblastoma compared to other tumor subtypes in The Cancer Genome Atlas (TCGA) Pan-Cancer Atlas dataset.

File Name: Supplementary Data 4
Description: Comparison of *ADORA1-3* and *ENTPD1/CD39* bulk mRNA expression in glioblastoma compared to other cancer subtypes in The Cancer Genome Atlas (TCGA) Pan-Cancer Atlas dataset.

File Name: Supplementary Data 5
Description: Demographic data for CNS tumors in the CD73 immunohistochemistry analysis

File Name: Supplementary Data 6
Description: CD73 expression in medulloblastoma stratified by histologic and molecular subtype

File Name: Supplementary Data 7
Description: CD73 expression by histologic region in adamantinomatous and papillary craniopharyngioma.

File Name: Supplementary Data 8
Description: CD73 expression in meningioma stratified by histologic subtype and W.H.O. grade.

File Name: Supplementary Data 9
Description: CD73 expression in CNS tumors stratified by recurrence status

File Name: Supplementary Data 10
Description: CD73 expression in IDH-wildtype glioblastoma stratified by *MGMT* promoter methylation status

File Name: Supplementary Data 11
Description: Metabolite standards used in the mass spectrometry analysis of glioblastoma

File Name: Supplementary Data 12
Description: Table of contents for the Source Data file, with the location of data for each figure panel in the manuscript.
