# Supplementary material for: Single cell spatial analysis reveals the topology of immunomodulatory purinergic signaling in glioblastoma
Source: Nat Commun. 2022 Aug 16;13:4814. doi: 10.1038/s41467-022-32430-w (PMC9381513; doi:10.1038/s41467-022-32430-w)
Supplement: Supplementary file 16 — Reporting Summary [file 41467_2022_32430_MOESM16_ESM.pdf]

nature portfolio

Corresponding author(s): Sandro Santagata
Last updated by author(s): 06/11/2022

Reporting Summary

Nature Portfolio wishes to improve the reproducibility of the work that we publish. This form provides structure for consistency and transparency in reporting. For further information on Nature Portfolio policies, see our Editorial Policies and the Editorial Policy Checklist.

Statistics

For all statistical analyses, confirm that the following items are present in the figure legend, table legend, main text, or Methods section.

n/a	Confirmed	
☐	☒	The exact sample size (*n*) for each experimental group/condition, given as a discrete number and unit of measurement
☐	☒	A statement on whether measurements were taken from distinct samples or whether the same sample was measured repeatedly
☐	☒	The statistical test(s) used AND whether they are one- or two-sided<br>*Only common tests should be described solely by name; describe more complex techniques in the Methods section.*
☐	☒	A description of all covariates tested
☐	☒	A description of any assumptions or corrections, such as tests of normality and adjustment for multiple comparisons
☐	☒	A full description of the statistical parameters including central tendency (e.g. means) or other basic estimates (e.g. regression coefficient) AND variation (e.g. standard deviation) or associated estimates of uncertainty (e.g. confidence intervals)
☐	☒	For null hypothesis testing, the test statistic (e.g. *F*, *t*, *r*) with confidence intervals, effect sizes, degrees of freedom and *P* value noted<br>*Give P values as exact values whenever suitable.*
☒	☐	For Bayesian analysis, information on the choice of priors and Markov chain Monte Carlo settings
☒	☐	For hierarchical and complex designs, identification of the appropriate level for tests and full reporting of outcomes
☐	☒	Estimates of effect sizes (e.g. Cohen's *d*, Pearson's *r*), indicating how they were calculated

Our web collection on statistics for biologists contains articles on many of the points above.

Software and code

Policy information about availability of computer code

Data collection	Immunofluorescence data were collected using the IN Cell 6000 Analyzer and Acquisition Software (v6.1 and v7.3), RareCyte CyteFinder II Imager and CyteFinder software (v3.11.024), and Deltavision Elite microscopes and SoftWoRx software (v7.0.0). The mass spectrometry imaging experiments were conducted using a 9.4 Tesla SolariX XR FT-ICR MS.
Data analysis	Commercial software: Matlab v2017, v2018a, v2019b, ImageJ (v1.53r), Microsoft Office 365 (Excel, Word), SCiLS Lab software (v2019c), MetaboAnalyst (v5.0), GraphPad Prism (v9.3.1).<br><br>Open source software: ASHLAR (v1.10.2) (https://github.com/labsyspharm/ashlar), MCMICRO (https://mcmicro.org/), ilastik (v1.3.3), python (v3.6), R (v4.2.0), including the Seurat (v4.1.1), ggplot2 (v3.3.6), ComplexHeatmap (v3.15), ABSOLUTE, and ggsignif packages.<br><br>Additional custom code used in this study have been deposited at:<br>Github: https://github.com/labsyspharm/cd73_coy_spatialcorrelation<br>Zenodo: https://zenodo.org/record/6628875#.YqJVfqjMJD8, DOI: 10.5281/zenodo.6628874

For manuscripts utilizing custom algorithms or software that are central to the research but not yet described in published literature, software must be made available to editors and reviewers. We strongly encourage code deposition in a community repository (e.g. GitHub). See the Nature Portfolio guidelines for submitting code & software for further information.

Data

Policy information about availability of data

All manuscripts must include a data availability statement. This statement should provide the following information, where applicable:

- Accession codes, unique identifiers, or web links for publicly available datasets
- A description of any restrictions on data availability
- For clinical datasets or third party data, please ensure that the statement adheres to our policy

Data used in the preparation of this manuscript are detailed in the Source Data file provided with the manuscript.

The processed cyclic immunofluorescence (CyCIF) single-cell data feature tables and mass spectrometry data for the adult glioma, pediatric glioma, and mouse brain datasets are deposited and available at the Synapse repository: https://www.synapse.org/#!Synapse:syn30803357

The publicly available adult glioblastoma single-cell RNA sequencing data (Neftel et al.) are available in the Broad Single Cell Portal (https://singlecell.broadinstitute.org/single_cell/study/SCP393/single-cell-rna-seq-of-adult-and-pediatric-glioblastoma#study-summary)

The publicly available adult glioma single-cell immune RNA-sequencing data (Pombo-Antunes et al.) are available in the Brain Immune Atlas (https://www.brainimmuneatlas.org/)

The publicly available developing brain single-cell RNA-sequencing data (Nowakowski et al.) are available in the UCSC Cell Browser (https://cells.ucsc.edu/?ds=cortex-dev)

The publicly available human microglia single-cell RNA-sequencing data (Sankowski et al.) are available in the human microglia browser (https://single-cell.shinyapps.io/human-microglia/)

The publicly available processed TCGA data are available at https://www.cbioportal.org/

The publicly available processed CPTAC/CBTTC pediatric glioma data are available at: http://pbt.cptac-data-view.org/

The publicly available TCGA GBM bulk full-length RNA-seq processed by the Piccolo's team82 are available at: https://osf.io/gqrz9/files

Mass spectrometry metabolite identities were cross-matched using the Metlin metabolite database (https://metlin.scripps.edu/landing_page.php?pgcontent=mainPage)

Remaining data are available within the Article, Source Data, or Supplementary Tables and Figures.

There are no restrictions on data access. Raw imaging data from cyclic immunofluorescence experiments are available from the corresponding authors on appropriate request. These files are large and may require significant computational pre-processing for final data analysis. The processes used by the authors are delineated in the Methods section and/or code repository (https://github.com/labsyspharm/cd73_coy_spatialcorrelation), but interested parties should first consider using processed counts tables available in the Synapse repository (https://www.synapse.org/#!Synapse:syn30803357).

Further information and requests for resources and reagents should be directed to and will be promptly fulfilled by the corresponding authors.

Field-specific reporting

Please select the one below that is the best fit for your research. If you are not sure, read the appropriate sections before making your selection.

☒ Life sciences ☐ Behavioural & social sciences ☐ Ecological, evolutionary & environmental sciences

For a reference copy of the document with all sections, see nature.com/documents/nr-reporting-summary-flat.pdf

Life sciences study design

All studies must disclose on these points even when the disclosure is negative.

Sample size

For single-cell RNA-sequencing analyses, multiple well-established public datasets were leveraged for analysis, including those generated and reported by Neftel et al. (n=28 human tumors), Pombo Antunes et al. (n=11 human tumors), Sankowski et al. (n=15 human brain specimens), and Nowakowski et al. (n=13 human brain specimens). For these datasets, we used all available samples for analysis.

Additional transcriptomic and copy-number analyses were performed using the large and well-established The Cancer Genome Atlas (TCGA) and Children's Brain Tumor Tissue Consortium (CBTTC) datasets. For these datasets, we used all available samples for analysis.

For immunohistochemistry and CyCIF imaging experiments, sample sizes were not pre-determined through statistical criteria, but a large number of specimens robustly exceeding the sample sizes from related literature on purine pathway protein expression in glioblastoma and single cell imaging were acquired and analyzed to provide adequate cellular, histologic, and genotypic sample diversity.
Immunohistochemistry experiments included several hundred human tumor specimens (n=604 total specimens), including nearly 200 adult

	human glioblastoma specimens for the primary adult CyCIF cohort, and nearly 100 tumors for the pediatric cohort. Clinicopathologic and genotypic analyses of the adult and pediatric cohorts showed that these groups contained a robust diversity of histologic and molecular phenotypes as well as a typical frequency of recurrent alterations, which provided additional confidence that the cohorts provided an accurate and robust representation of the biological diversity of human adult glioblastoma and pediatric high-grade glioma.
Data exclusions	For tissue microarray data (CyCIF, immunohistochemistry), tissue QC was performed by a pathologist and a small minority of cases in which the tissue core was insufficient for analysis (folded, minimal tissue, absent tissue) were excluded, blinded to experimental parameters such as marker expression, diagnosis, and clinical data. Whenever data was filtered to select a sub-population for analysis or a figure (e.g., primary IDH-wildtype glioblastoma) the procedure is detailed in the text, figure legend, or methods section, and additional sub-cohort data is described (see supplementary tables). For single-cell immunofluorescence data, QC was performed as described in the methods section and associated references.
Replication	All experimental analyses, including spatial statistics, single-cell protein and mRNA expression analysis, clinical outcomes analysis, and tissue analysis were technically reproducible. For immunohistochemistry, mass spectrometry, and CyCIF experiments the staining and imaging were performed once for each specimen given the technical complexity of the assays and large cohorts of biologically independent human tumor specimens involved. For CyCIF experiments, tissue microarrays included multiple replicate cores for each tumor specimen with similar staining patterns between replicates. The main experimental findings were cross-validated using multiple orthogonal analytic methods (single-cell RNA-sequencing, multiplexed immunofluorescence, immunohistochemistry, mass spectrometry).
Randomization	Randomization was not relevant or necessary for the experiments and analysis performed. The study involves retrospective analysis of large cohorts of human tumor specimens and/or public databases. No prospective patient enrollment was involved in the study. Clinical outcomes analyses are based on retrospective analysis of de-identified clinical data.
Blinding	When visual tissue analyses were performed (e.g., histologic tissue QC, IF/IHC evaluation and QC, CD73 IHC scoring by a pathologist), relevant investigators (S.C., S.S.) were blind to the precise diagnosis (e.g., IDH-mutant vs. IDH-wildtype, H3K27M-mutant diffuse midline glioma vs. pediatric HGG, NOS) and clinical (e.g., patient demographics, PFS, OS), molecular (e.g., IDH1/2-status, EGFR-status, MGMT-status, MMR-status, etc.), and other relevant data to avoid bias in assessment of tissue adequacy and scoring.<br><br>Some investigators (S.W., J.R.L.) performing the initial single-cell, spatial statistics, and outcome analyses using CyCIF imaging data were not involved in the initial tumor cohort selection or visual tissue analysis, and were generally blind to source tissue of cells except where necessary during the analysis of single-cell imaging data to further avoid potential bias in the processing of single-cell data and application of analytic methods to this data.

Reporting for specific materials, systems and methods

We require information from authors about some types of materials, experimental systems and methods used in many studies. Here, indicate whether each material, system or method listed is relevant to your study. If you are not sure if a list item applies to your research, read the appropriate section before selecting a response.

Materials & experimental systems

n/a	Involved in the study	
☐	☒	Antibodies
☒	☐	Eukaryotic cell lines
☒	☐	Palaeontology and archaeology
☒	☐	Animals and other organisms
☐	☒	Human research participants
☒	☐	Clinical data
☒	☐	Dual use research of concern

Methods

n/a	Involved in the study	
☒	☐	ChIP-seq
☒	☐	Flow cytometry
☒	☐	MRI-based neuroimaging

Antibodies

Antibodies used

anti-Arginase-1 (D4E3M) Cell Signaling Technology 66297 dilution 1:100, AB_2799705
anti-CA IX (H-11) Santa Cruz sc-365900 AF488 dilution 1:100, AB_10846466
anti-CD11b (C67F154) eBioscience 53-0196-82 dilution 1:100, AB_2637195
anti-CD14 [EPR3653] Abcam ab196169 dilution 1:800, AB_2890135
anti-CD163 [EPR14643-36] Abcam ab218293 1:100, AB_2889155
anti-CD206 (D-1) Santa Cruz sc-376108 AF488 dilution 1:100, AB_10987732
anti-CD24 [SN3] Abcam ab30350 dilution 1:200, AB_726282
anti-CD3 [CD3-12] Abcam ab11089 dilution 1:500, AB_2889189A
anti-CD31 [EPR3094] Abcam ab218582 dilution 1:500, AB_2857973
anti-CD39 [EPR20627] Abcam ab236038 dilution 1:100, n/a
anti-CD4 (N1UG0) eBioscience 41-2444-82 dilution 1:100, AB_2573602
anti-CD40 (D8W3N) Cell Signaling Technology 40868 dilution 1:100, AB_2799188
anti-CD44 [EPR1013Y] Abcam ab194987 dilution 1:100, n/a
anti-CD44 (156-3C11) Cell Signaling Technology 8724 dilution 1:200, AB_10829611
anti-CD45 (HI30) BioLegend 304039 dilution 1:200, AB_2562057
anti-CD68 (D4B9C) Cell Signaling Technology 79594S dilution 1:200, AB_2799935
anti-CD73 [EPR6115], Abcam ab124725 dilution 1:200, AB_10976033

anti-CD73 [EPR6114], Abcam ab133582 dilution 1:200, n/a
anti-CD73 (4G6E3), Abcam ab202122 dilution 1:200, AB_2858258
anti-CD73 (D7F9A), Cell Signaling Technology 1:200, AB_2716625
anti-CD8a (AMC908) eBioscience 50-0008-80 dilution 1:200, AB_2574148
anti-EGFR (D38B1) Cell Signaling Technology 5616 dilution 1:200, AB_10691853
anti-FOXO3A (D19A7) Cell Signaling Technology 14592 dilution 1:200, AB_2798529
anti-FOXP3 (236A/E7) eBioscience 41-4777-82 dilution 1:200, AB_2573609
anti-GFAP (GA5) eBioscience 41-9892-82 dilution 1:100, AB_2573656
anti-GLUT1 [EPR3915] Abcam ab195020 dilution 1:100, AB_2783877
anti-HIF-1a (D1S7W) Cell Signaling Technology 52496 dilution 1:100, AB_2799414
anti-IBA1 [EPR6136(2)] Abcam ab195031 dilution 1:500, AB_2889157
anti-iNOS [EPR16635] Abcam ab209594 dilution 1:100, AB_2889252
anti-Ki67 (D3B5) Cell Signaling Technology 11882 dilution 1:400, AB_2687824
anti-MeCP2 (D4F3) Cell Signaling Technology 34113 dilution 1:200, AB_2799046
anti-Mouse Invitrogen A-21235 dilution 1:1000, AB_2535804
anti-NeuroD1 Novus NBP2-98697 AF488 dilution 1:200, n/a
anti-Olig-2 Millipore AB9610-AF555 dilution 1:200, AB_570666
anti-p21/CDKN1a (12D1) Cell Signaling Technology 8587 dilution 1:200, AB_10892861
anti-p53 [E26] Abcam ab224942 dilution 1:400, AB_2889206
anti-PD1 [EPR4877(2)] Abcam ab201825 dilution 1:200, AB_2728811
anti-PDGFRa (D13C6) Cell Signaling Technology 8893 dilution 1:200, AB_2797679
anti-PD-L1 (E1L3N) Cell Signaling Technology 15005 dilution 1:200, AB_2728832
anti-pH2AX (2F3) BioLegend 613412 dilution 1:100, AB_2616871
anti-PU.1 (G148-74) BD Biosciences 554268 dilution 1:200, AB_395335
anti-PU.1 (9G7) Cell Signaling Technology 81886 dilution 1:100, AB_2799984
anti-Rabbit Invitrogen A-11008 dilution 1:1000, AB_143165
anti-Rabbit (Labeled Polymer HRP) Dako Cytomation Envision System K4011
anti-Rat Invitrogen A-21434 dilution 1:1000, AB_141733
anti-SOX2 [EPR3131] Abcam ab196637 dilution 1:100, n/a
anti-SOX2 (D6D9) Cell Signaling Technology 5179 dilution 1:200, AB_10828439
anti-Survivin (71G4B7) Cell Signaling Technology 2810 dilution 1:200, AB_10691462

Validation

Antibody validation was performed by 1) comparing signal between positive and negative tissue samples, including control tonsil tissue 2) comparison between IF and IHC staining 3) sub-cellular localization (e.g., membranous, cytoplasmic, nuclear), 3) marker co-localization 4) lack of co-localization with markers known not to co-localize.

Additionally, multiple antibodies used in this study have been previously used in multiple projects by our group providing longitudinal information on specificity and testing of expected patterns of expression, including:

anti-CD11b (C67F154) eBioscience 53-0196-80 dilution 1:200, AB_2637195
anti-CD11b (C67F154) eBioscience 53-0196-82 dilution 1:100, AB_2637195
anti-CD14 [EPR3653] Abcam ab196169 dilution 1:800, AB_2890135
anti-CD163 [EPR14643-36] Abcam ab218293 1:100, AB_2889155
anti-CD45 (HI30) BioLegend 304039 dilution 1:200, AB_2562057
anti-CD68 (D4B9C) Cell Signaling Technology 79594S dilution 1:200, AB_2799935
anti-CD8a (AMC908) eBioscience 50-0008-80 dilution 1:200, AB_2574148
anti-FOXP3 (236A/E7) eBioscience 41-4777-82 dilution 1:200, AB_2573609
anti-GFAP (GA5) eBioscience 41-9892-82 dilution 1:100, AB_2573656
anti-IBA1 [EPR6136(2)] Abcam ab195031 dilution 1:500, AB_2889157
anti-PD1 [EPR4877(2)] Abcam ab201825 dilution 1:200, AB_2728811
anti-PD-L1 (E1L3N) Cell Signaling Technology 15005 dilution 1:200, AB_2728832
Du Z, et al. Nat Protoc. 2019 Oct;14(10):2900-2930. doi: 10.1038/s41596-019-0206-y.

anti-CD206 (D-1) Santa Cruz sc-376108 AF488 dilution 1:100, AB_10987732
anti-CD31 [EPR3094] Abcam ab218582 dilution 1:500, AB_2857973
anti-CD40 (D8W3N) Cell Signaling Technology 40868 dilution 1:100, AB_2799188
anti-GLUT1 [EPR3915] Abcam ab195020 dilution 1:100, AB_2783877
Nirmal et al. Cancer Discov. 2022 Jun 2;12(6):1518-1541. doi: 10.1158/2159-8290.CD-21-1357

anti-Ki67 (D3B5) Cell Signaling Technology 11882 dilution 1:400, AB_2687824
Gaglia et al. Nat Cell Biol. 2022 Mar;24(3):316-326. doi: 10.1038/s41556-022-00860-9.)

For CD73, to further confirm antibody specificity cyclic immunofluorescence was performed with multiple distinct antibody clones (EPR6114, EPR6115, 4G6E3, D7F9A) on a tissue microarray of glioblastoma cases and pixel-level signal correlation was analyzed, in accordance with recommendations published in "Qualifying antibodies for image-based immune profiling and multiplexed tissue imaging" Nat Protoc. 2019 Oct;14(10):2900-2930. PMID: 31534232. All anti-CD73 antibody clones showed a strong correlation in signal (Figure S10a,b).

Additionally, vendor and literature knockout experiments with each clone were reviewed to further support the specificity of binding.

For EPR6114, antibody specificity was validated by western blot on NT5E/CD73 knockout human A-431 cells by the vendor (Abcam) (https://www.abcam.com/cd73-antibody-epr6114-ab133582.html)
For 4G6E3, antibody specificity was validated by western blot on NT5E/CD73 knockout human A-375 cells by the vendor (Abcam) (https://www.abcam.com/cd73-antibody-4g6e3-ab202122.html)
For D7F9A, antibody specificity was supported by siRNA/shRNA knockdown experiments in multiple literature reports (https://pubmed.ncbi.nlm.nih.gov/33086655/, https://pubmed.ncbi.nlm.nih.gov/33609609/, https://pubmed.ncbi.nlm.nih.gov/28158983/)

Human research participants

Policy information about studies involving human research participants

Population characteristics	Population characteristics analyzed included patient age, sex, race, diagnosis, recurrence status, surgery type (gross total resection, subtotal resection, etc.), tumor location, tumor size, pre-operative steroid administration status, progression, death, progression-free survival (PFS), overall survival (OS).<br><br>Genotypic information analyzed for glioma and other CNS specimens from the BWH cohort included targeted-exome sequencing (447-gene panel, oncopanel), chromosomal copy-number analysis (array comparative genomic hybridization), and MGMT promoter methylation analysis which were obtained during routine clinical diagnostic practice in a CLIA-approved clinical laboratory at BWH.<br><br>Genotypic information analyzed for the CHOP/CBTTC pediatric glioma cohort included whole-genome sequencing.
Recruitment	Our research complies with all relevant ethical regulations and was reviewed and approved by the Institutional Review Boards (IRB) at Brigham and Women's Hospital (BWH), Harvard Medical School (HMS), Dana Farber Cancer Institute (DFCI), and Children's Hospital of Philadelphia (CHOP).<br><br>Discarded human formalin fixed paraffin embedded (FFPE) tissue samples were used after diagnosis under excess tissue discarded tissue protocol 2018P001627 (reviewed and managed by the Mass General Brigham Institutional Review Board) which waives the requirement for patient consent. Adult glioblastoma samples and pediatric high-grade glioma samples subjected to genomic analysis were used after patient consent had been obtained under Dana-Farber Cancer Institute IRB protocol 10-417 and Children's Hospital of Philadelphia IRB protocol 19-016112. For pediatric patients, consent for genomic analysis was obtained from the legally authorized representatives/guardians. The study is compliant with all relevant ethical regulations regarding research involving human tissue specimens. The authors identified no potential biases in specimen selection that may have affected the experimental results.
Ethics oversight	Our research complies with all relevant ethical regulations and was reviewed and approved by the Institutional Review Boards (IRB) at Brigham and Women's Hospital (BWH), Harvard Medical School (HMS), Dana Farber Cancer Institute (DFCI), and Children's Hospital of Philadelphia (CHOP). For the CPTAC/CBTTC cohort, all subjects consented to tissue and data collection through CBTTC Institutional Review Board–approved protocols.

Note that full information on the approval of the study protocol must also be provided in the manuscript.
